# Use and Perceived Helpfulness of Different Intervention Strategies in Myalgic Encephalomyelitis/Chronic Fatigue Syndrome and Depression

**DOI:** 10.3390/jcm15020849

**Published:** 2026-01-20

**Authors:** Marie Celine Dorczok, Nilufar Mossaheb, Gloria Mittmann, Marina F. Thomas, Lucie Bartova, Beate Schrank, Verena Steiner-Hofbauer

**Affiliations:** 1 Research Centre Transitional Psychiatry at the University Hospital Tulln—NOE LGA, Faculty of Medicine, Karl Landsteiner University, 3430 Tulln, Austria; 2 Division of Social Psychiatry, Department of Psychiatry and Psychotherapy, Medical University of Vienna, 1090 Vienna, Austria; 3 Comprehensive Center for Clinical Neurosciences and Mental Health, Medical University of Vienna, 1090 Vienna, Austria; 4 Clinical Division of General Psychiatry, Department of Psychiatry and Psychotherapy, Medical University of Vienna, 1090 Vienna, Austria; 5 Department of General Psychiatry, Vienna Health Association Clinic Ottakring, 1160 Vienna, Austria

**Keywords:** myalgic encephalomyelitis/chronic fatigue syndrome, depression, dietary supplementation, symptom management, multivariate analysis, patient-reported outcomes

## Abstract

**Background:** Patients with myalgic encephalomyelitis/chronic fatigue syndrome (ME/CFS) or depression both frequently report debilitating exhaustion, yet the two conditions differ in their etiological and diagnostic clarity, and clinical management. This study aimed to examine differences in the use and perceived helpfulness of a broad range of conventional treatments and complementary interventions, including nutritional approaches, between patients with ME/CFS and depression. **Methods:** A cross-sectional online survey was conducted in 2024. A total of 819 participants self-identified as having either ME/CFS (*n* = 576) or depression (*n* = 243). Participants (80% female) reported their use and perceived helpfulness of 52 treatments and interventions, encompassing behavioral therapies, medications, and dietary supplements. Group differences were examined using multivariate analyses of variance and covariance (MANOVA/MANCOVA). Open-ended responses were analyzed descriptively using thematic grouping and frequency counts. **Results:** Participants with depression most commonly reported the use of psychotherapy (*M* = 2.49, *SD* = 1.00) and antidepressant medication (*M* = 2.44, *SD* = 2.30), and they rated fewer interventions as helpful compared to participants with ME/CFS. In contrast, participants with ME/CFS reported a significantly broader engagement with diverse intervention modalities, particularly pacing (*M* = 2.73, *SD* = 0.80) and dietary supplements (*M* = 2.43, *SD* = 1.09), and perceived many of them as helpful. Group differences remained significant after controlling for age, gender, and whether treatment was medically recommended. Supplements targeting energy metabolism (e.g., CoQ10, NADH) were especially favored among ME/CFS participants. **Conclusions:** Findings suggest that participants with ME/CFS tend to adopt an exploratory and expansive intervention approach, potentially reflecting the lack of standardized guidelines and limited effectiveness of available treatment options. Participants with depression, in contrast, appeared to follow more guideline-concordant, evidence-based treatment pathways. Taken together, the findings point to a need for further development and evaluation of empirically supported, patient-centered treatment and intervention strategies for ME/CFS and suggest differences in clinical care structures between ME/CFS and depression.

## 1. Introduction

Myalgic encephalomyelitis/chronic fatigue syndrome (ME/CFS) is a severe chronic condition characterized by post-exertional malaise (PEM), persistent non-restorative fatigue, and a range of debilitating symptoms, including cognitive impairment (“brain fog”), sleep disturbances, pain, and orthostatic intolerance [1,2]. Prevalence estimates range between 0.2–0.8% in the general population, with consistently higher rates reported among women and individuals in middle adulthood [3]. ME/CFS is associated with substantial functional impairment [4].

Despite increasing evidence implicating immune dysregulation, metabolic alterations, and autonomic dysfunction, the pathophysiological mechanisms of ME/CFS have not yet been fully elucidated [5,6,7,8,9,10], and no validated biomarkers or curative treatments have been established to date [11]. Clinical management, therefore, primarily focuses on symptom-oriented and supportive approaches, such as pacing [12,13], off-label pharmacological treatments (e.g., phytotherapy [14]; low-dose naltrexone [15,16]; aripiprazole [17]; ketamine [18]), or dietary supplements (DS) [19,20]. Due to diagnostic uncertainty, limited availability of specialized care, and the absence of standardized evidence-based treatment pathways [21,22], many patients with ME/CFS rely on self-directed coping strategies and a broad range of symptom management practices [23,24].

Symptom overlap with other disorders further complicates diagnosis and treatment decisions. In particular, fatigue, cognitive difficulties, and sleep disturbances are core features of both ME/CFS and depression, rendering differential diagnosis challenging [25,26,27,28]. Moreover, depression frequently co-occurs with ME/CFS, as is common in many chronic and disabling somatic conditions, contributing to increased disease burden and complexity of care [29,30].

Depression is one of the most common mental disorders worldwide. According to the World Health Organization, an estimated 5.7% of adults globally suffer from depressive disorder, corresponding to more than 330 million affected individuals, with significantly higher prevalence among women compared to men [31]. It is characterized by persistent low mood, anhedonia, cognitive impairments, reduced energy and drive, and somatic symptoms including vegetative hyperarousal [32,33]. Unlike ME/CFS, depression benefits from well-established diagnostic frameworks and guideline-based treatments, including psychotherapy and pharmacotherapy, whereby selective serotonin reuptake inhibitors (SSRIs) and serotonin–norepinephrine reuptake inhibitors (SNRIs) represent the recommended first-line antidepressants [34,35]. Meta-analytic data indicate that antidepressant medications demonstrate small to moderate effect sizes over placebo (Hedges’ g = 0.24–0.30) in the treatment of depression [36]. Additionally, a meta-analysis of randomized controlled trials has found that cognitive behavioral therapy (CBT) for depressive symptoms yields moderate effect sizes, with summary effect estimates in the range of Hedges’ g = 0.56–0.63 compared with control conditions [37].

### 1.1. Coping Strategies and Treatment-Seeking Behavior in ME/CFS and Depression

Beyond guideline-based care, a notable proportion of patients with depression also engage in complementary or self-directed approaches, either to augment standard treatments or to address residual symptoms and unmet needs [38,39,40,41,42]. Systematic reviews indicate that up to 50% of individuals with depressive disorders use complementary and alternative medicine (CAM), including nutraceuticals (e.g., omega-3 fatty acids, probiotics), meditation, yoga, and bright-light therapy, albeit with low to variable quality evidence and few rigorous randomized controlled trials supporting clinical efficacy [38,43,44].

Similarly, patients with ME/CFS frequently report the use of non-pharmacological strategies and complementary interventions, including nutraceuticals, mind–body techniques, and lifestyle-based modifications [45,46,47,48,49,50,51]. Systematic reviews of RCTs in ME/CFS document a large diversity of both pharmacological and non-pharmacological interventions but conclude that no consistently effective therapy has been established, and many approaches yield mixed or inconclusive results [52,53]. Classical large-scale trials such as the PACE study initially suggested moderate benefits for graded exercise therapy (GET) and CBT [12], but these findings have been widely criticized on methodological grounds and are no longer broadly recommended [54,55]. GET in particular is now contraindicated in many clinical guidelines due to potential worsening symptoms, while CBT may serve as an adjunctive coping support rather than a curative treatment [56,57]. Evidence for specific DS strategies in ME/CFS remains inconclusive, with some small RCTs combining coenzyme Q10 and NADH showing improvements in fatigue and related outcomes but large, high-quality trials lacking definitive findings [51].

Despite heterogeneity and limited evidence, such coping and self-directed strategies are actively pursued by many patients with ME/CFS and depression alike [38,58]. This pattern may reflect efforts to regain agency in managing a poorly understood condition, perceived limitations within healthcare systems, and the influence of informal recommendations within patient communities [59,60,61]. Importantly, patients’ perceptions of treatment helpfulness are shaped not only by objective symptom change but also by illness beliefs, diagnostic recognition, expectations, and prior treatment experiences, all of which influence adherence, trust, and engagement with different interventions [62].

### 1.2. Study Aim

Against this background, the present study compares the use and perceived helpfulness of a broad range of conventional, complementary, and self-directed interventions among self-identified patients with ME/CFS and depression. By examining two conditions that share substantial symptom overlap yet differ markedly in diagnostic recognition, availability of evidence-based treatments, and clinical care structures, this study seeks to elucidate how structural, experiential, and psychosocial factors shape patterns of intervention use and perceived helpfulness.

## 2. Materials and Methods

### 2.1. Recruitment and Procedure

This study represents a sub-project within the broader research initiative FATIGEAT, which investigates the phenomenology and help-seeking behavior of patients diagnosed with ME/CFS and depression. The data collection was carried out between May and December 2024 at Karl Landsteiner University of Health Sciences, Krems, Austria, and Medical University of Vienna, Austria.

A cross-sectional online survey was developed and administered via the SoSciSurvey platform Version 3.5.02 (SoSciSurvey GmbH, Munich, Germany) [63]. Participants were recruited through psychiatric and psychotherapeutic inpatient units and day clinics, as well as through private psychiatric and psychotherapeutic practices in Vienna and Tulln, Austria, but also through social media channels to reach those who are housebound or have limited access to medical care. The survey was accessible via a link or QR code and could be completed using any internet-enabled device. Participants were not offered financial compensation.

Eligibility criteria included an age range of 18 to 70 years and a self-reported diagnosis of either ME/CFS or depression. Participation was voluntary and anonymous, with informed consent obtained prior to enrollment. Group assignment was based on self-reported diagnosis of ME/CFS or depression. To characterize symptom profiles, the survey additionally included the DASS-21 (for depressive symptom severity) [64] and the DSQ-PEM (to assess PEM as the cardinal feature of ME/CFS) [65]. The corresponding results are reported elsewhere [Dorczok et al., manuscript under review]. No further diagnostic verification beyond self-report was undertaken. To ensure a clear and unambiguous separation between the self-identified diagnostic groups, recruitment for each group was conducted independently using tailored flyers, each containing unique QR codes and access links. These directed participants to two technically separate, but content-identical, online surveys, allowing for subsequent direct comparison between ME/CFS and depression groups using equivalent survey instruments. The estimated completion time for the survey was approximately 30 to 40 min. To accommodate participants with cognitive or physical impairments, the option to pause and resume the survey later was provided. Only fully completed questionnaires were included in the final analysis.

### 2.2. Materials

The survey contained single-item questions on help-seeking behaviors, including previous treatment and intervention attempts, as well as their perceived helpfulness. In more detail, participants were asked about their intervention history, including current and past use of interventions in general, medication, and dietary supplements for symptoms related to their condition, either ME/CFS or depression. Duration of interventions as well as self-reported helpfulness were assessed on 6-point Likert scales (duration of intervention: 0 = never; or 1 = tried once; 2 = 1–3 months; 3 = 4–6 months; 4 = 7–12 months; 5 = longer than 12 months; helpfulness of intervention: 0 = never tried; 1 = not at all helpful to 5 = very helpful), respectively. Additionally, open-text boxes were provided for further information if needed. The survey also inquired whether interventions were used following a doctor’s prescription or on the participant’s own initiative.

### 2.3. Statistical Analyses

Statistical analyses were performed using SPSS 29.0.1.0 (IBM, Archmond, NY, USA) [66]. Descriptive statistics were calculated for sociodemographic variables and reasons for DS intake. For sociodemographic data, group comparisons were conducted using non-parametric Mann–Whitney U tests for continuous variables and chi-square tests for categorical variables.

For descriptive purposes, intervention use was additionally dichotomized to estimate prevalence rates. Scores of ≥1 on the use scale were classified as indicating current or recent use of the respective intervention, whereas a score of 0 indicated no use. The same dichotomization approach was applied to perceived helpfulness, with scores of ≥1 indicating that an intervention was perceived as helpful. Prevalence estimates for both use and perceived helpfulness are presented in bar charts to enhance clarity and facilitate comparison across interventions and diagnostic groups.

To examine group differences between participants with ME/CFS and those with depression, data from both surveys were merged, and a series of multivariate analyses of variance (MANOVA) was conducted. The independent variable in all models was diagnostic group (ME/CFS vs. depression). The dependent variables were mean ratings of intervention use and perceived helpfulness. Participants who had not used an intervention were not asked about its helpfulness; their rating was coded as 0 (“never tried, so not assessable”). MANOVAs were conducted using the continuous mean ratings rather than dichotomized variables, as dichotomization would have resulted in a substantial loss of information. This approach was chosen to retain variability in frequency and intensity of use and perceived helpfulness, and to adequately reflect the multivariate nature of treatment engagement, given that many participants reported concurrent use of multiple interventions.

Separate MANOVAs were conducted for three conceptually distinct domains, i.e., interventions, medication, and DS advertised for fatigue symptoms, with use and perceived helpfulness as dependent variables each. Prior to analysis, assumptions of multivariate normality, linearity, and homogeneity of variance-covariance matrices were examined. Given substantial variance heterogeneity between groups, Pillai’s trace was used as a robust test statistic. Although normality was not fully met for some helpfulness ratings due to zero responses, the robustness of the applied statistics mitigates this concern. Significant multivariate effects were followed by univariate ANOVAs and Bonferroni-adjusted post hoc comparisons to identify specific group differences. In addition, a post hoc power analysis was conducted to evaluate whether the achieved sample size provided sufficient statistical power to detect the observed group differences. The analysis was based on the effect sizes (partial η^2^) obtained from the univariate ANOVAs. Statistical significance was set at *p* < 0.05.

Two multivariate analyses of covariance (MANCOVA) were conducted to examine group differences (ME/CFS vs. depression) in DS use and perceived helpfulness, controlling for age, gender, and whether DS use was medically recommended or self-initiated.

For clarity, the eight most relevant variables based on significance and effect size are presented. Data visualizations were created using Microsoft Excel (Microsoft, Redmond, WA, USA) [67]. Open-text responses were brief and keyword-based; thus, they were grouped thematically and analyzed descriptively by frequency of mentions within each group. Responses were translated from German to English using DeepL Version 25.8.2 (DeepL SE, Cologne, Germany) [68].

### 2.4. Ethical Approval

Ethical approval was granted by the Committee for Scientific Integrity and Ethics of Karl Landsteiner University of Health Sciences (Reference Number 1088/2023) and by the Ethics Committee of the Medical University of Vienna (Reference Number 1473/2024).

## 3. Results

Between May and December 2024, a total of 3637 participants accessed the online survey (2160 for ME/CFS and 1477 for depression). The final data set for analyses included 819 complete observations, comprising 576 participants with self-reported ME/CFS and 243 participants with self-reported depression. The response rate was 22.5%.

The overall sample was predominantly female (80%), with higher proportions of women in the ME/CFS group (89%) compared to the depression group (78%). Participants with ME/CFS were older on average (mean [*M*] = 46.37, standard deviation [*SD*] = 11.68) than those with depression (*M* = 38.37, *SD* = 13.54). BMI was comparable across both groups (ME/CFS: *M* = 25.8, *SD* = 6.14; depression: *M* = 25.78, *SD* = 6.86), and the duration of fatigue symptoms was generally longer in the ME/CFS group (*M* = 59.38 months, median [Mdn] = 31.00 months; *SD* = 67.95) than among those with depression (*M* = 49.96 months, Mdn = 26.00; *SD* = 59.35 months). Table 1 provides an overview of key sociodemographic characteristics.

### 3.1. Use of Interventions

**Figure 1:**
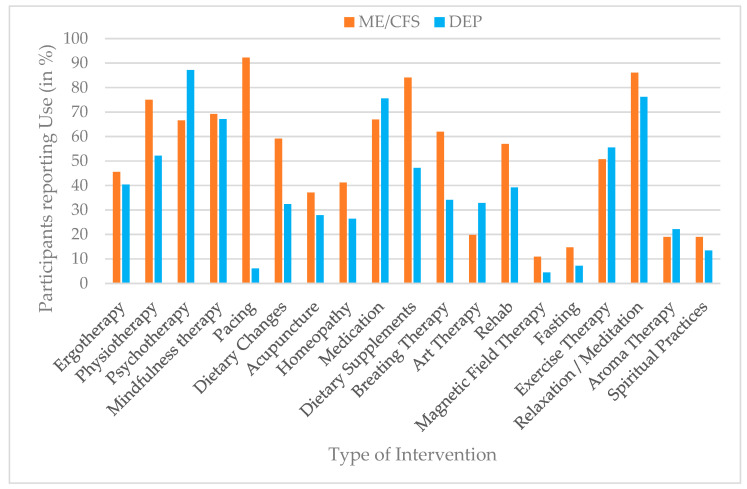
Prevalence of intervention use (%) by group (ME/CFS vs. depression), based on dichotomized use ratings (use vs. no use).

Participants with ME/CFS reported higher use of pacing (92%), dietary supplements (84%), and dietary changes (59%) compared to participants with depression, whereas psychotherapy (87%) and medication (76%) were more frequently used in the depression group. Mindfulness-based approaches (ME/CFS = 69%; depression = 67%) and relaxation/meditation (ME/CFS = 86%; depression = 76%) showed comparatively high use in both groups, though rates were consistently higher among ME/CFS participants. Several interventions, including magnetic field therapy and fasting, were used only by a small minority of participants across both diagnostic groups (<15%). Prevalences for intervention use are presented in detail in Figure 1.

A MANOVA confirmed statistically significant differences between the ME/CFS and depression groups in their reported use across the 19 interventions, F (19, 799) = 88.71, *p* < 0.001, Pillai’s trace = 0.678, partial η^2^ = 0.68, indicating a large multivariate effect. Box’s M test was significant (*M* = 559.42, F (190, 727,090) = 2.86, *p* < 0.001), yet the use of Pillai’s trace ensures robustness against unequal covariances.

Univariate follow-up analyses showed the largest effect for pacing, which was reported more frequently by ME/CFS participants (*M* = 2.73, *SD* = 0.80) than by participants with depression (*M* = 0.16, *SD* = 0.62), F (1, 817) = 2022.89, *p* < 0.001, partial η^2^ = 0.71. DS was also used more often by the ME/CFS group (*M* = 2.43, *SD* = 1.09) compared to the depression group (*M* = 1.22, *SD* = 1.34), F = 184.14, *p* < 0.001, partial η^2^ = 0.18. In contrast, participants with depression reported higher use of psychotherapy (*M* = 2.49, *SD* = 1.00 vs. ME/CFS: *M* = 1.81, *SD* = 1.34, η^2^ = 0.06) and medication (*M* = 2.20, *SD* = 1.26 vs. ME/CFS: *M* = 1.88, *SD* = 1.35, η^2^ = 0.01). Further significant group differences were found for breathing therapy, dietary changes, physiotherapy, and relaxation/meditation (all *p* < 0.001, partial η^2^ = 0.060–0.097), as well as significant group effects with very small effect sizes for acupuncture, mindfulness therapy, art therapy, homeopathy, magnetic field therapy, rehabilitation, and fasting (all *p* < 0.01, partial η^2^ = 0.008–0.031). By contrast, use of spiritual practices (*p* = 0.078), ergotherapy (*p* = 0.270), and aromatherapy *(p* = 0.817) did not significantly differ between groups. A detailed summary of these results is provided in Table 2.

A post hoc power analysis based on a small effect size (Cohen’s f = 0.11) indicated that the achieved sample size provided adequate statistical power (1 − β = 0.88) to detect group differences.

Additionally, the most common responses to the open-ended question regarding other non-listed interventions were osteopathy, traditional Chinese medicine, and cold and heat therapies.

### 3.2. Perceived Helpfulness of Interventions

**Figure 2:**
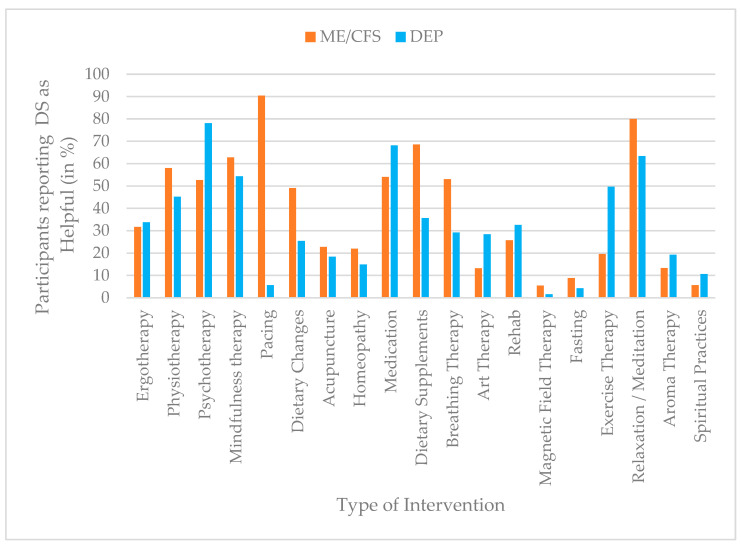
Prevalence of intervention helpfulness (%) by group (ME/CFS vs. depression), based on dichotomized helpfulness ratings (helpful vs. not helpful).

Participants with ME/CFS reported higher perceived helpfulness for several interventions, most notably pacing (90%), relaxation/meditation (80%), DS (69%), mindfulness therapy (63%), and physiotherapy (58%). In contrast, participants with depression most frequently rated psychotherapy (78%), medication (68%), relaxation/meditation (63%), exercise therapy (50%), and mindfulness therapy (54%) as helpful. Marked group differences were observed for pacing (ME/CFS = 90%; depression = 6%) and exercise therapy (ME/CFS = 20%; depression = 50%). Figure 2 provides an overview of the reported prevalences of intervention helpfulness.

A MANOVA confirmed statistically significant differences between the ME/CFS and depression groups in their helpfulness ratings across interventions, F (19, 799) = 88.53, *p* < 0.001, Pillai’s trace = 0.678, partial η^2^ = 0.68, indicating a large multivariate effect. Box’s M test was significant (*M* = 583.39, F (190, 727,090) = 2.98, *p* < 0.001), but the use of Pillai’s trace mitigates concerns regarding unequal covariances.

Univariate follow-up analyses showed the largest effect for pacing, which ME/CFS participants rated as substantially more helpful (*M* = 3.50, *SD* = 1.22) than participants with depression (*M* = 0.22, *SD* = 0.90), F (1, 817) = 1423.71, *p* < 0.001, partial η^2^ = 0.64. Other notable differences included higher helpfulness ratings for DS among ME/CFS participants (*M* = 1.92, *SD* = 1.93) compared to those with depression (*M* = 1.00, *SD* = 1.66), F (1, 817) = 184.14, *p* < 0.001, partial η^2^ = 0.18. In contrast, medication was perceived as more helpful by participants with depression (*M* = 2.60, *SD* = 1.79) compared to ME/CFS participants (*M* = 1.47, *SD* = 1.90), F (1, 817) = 66.19, *p* < 0.001, η^2^ = 0.08. Similarly, psychotherapy received higher helpfulness ratings from the depression group (*M* = 3.14, *SD* = 1.63) than from the ME/CFS group (*M* = 2.08, *SD* = 1.90), F (1, 817) = 57.15, *p* < 0.001, η^2^ = 0.07. Further significant group effects were found for exercise therapy, breathing therapy, dietary changes, and art therapy (all *p* < 0.001, η^2^ = 0.034–0.061), as well as smaller effects for physiotherapy, relaxation/meditation, acupuncture, homeopathy, and magnetic field therapy (all *p* < 0.01, η^2^ = 0.010–0.026). In contrast, several interventions were perceived similarly across groups, reflected in non-significant differences (all *p* > 0.05). This applied to mindfulness therapy, aromatherapy, spiritual practices, rehabilitation, ergotherapy, and fasting. A full overview of the results is provided in Table 3.

### 3.3. Use of Medication

**Figure 3:**
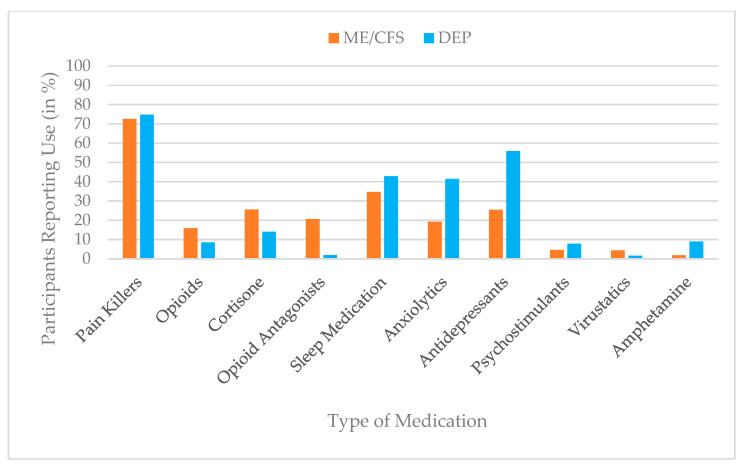
Prevalence of medication use (%) by group (ME/CFS vs. depression), based on dichotomized use ratings (use vs. no use).

Painkillers were the most commonly reported medication in both groups (ME/CFS: 73%; depression: 75%). Participants with depression reported markedly higher use of antidepressants (depression = 56%; ME/CFS = 26%) and anxiolytics (depression = 42%; ME/CFS = 19%), whereas participants with ME/CFS more frequently used cortisone (ME/CFS = 26%; depression = 14.0%) and opioid antagonists (ME/CFS = 21%; depression = 2%). Use of sleep medication was common in both groups but higher in depression (depression = 43%; ME/CFS = 35%), while all other medication classes showed low prevalence (<10%). An overview of medication use prevalence is shown in Figure 3.

A MANOVA confirmed a statistically significant multivariate group effect in medication use between ME/CFS and depression, F (10, 808) = 21.38, *p* < 0.001, Pillai’s trace = 0.209, partial η^2^ = 0.21. Box’s M test was significant (*M* = 1333.31, F (55, 761,859) = 23.85, *p* < 0.001), but the use of Pillai’s trace reduces concerns related to covariance heterogeneity.

Univariate follow-up ANOVAs revealed the largest group differences for antidepressants, with participants in the depression group reporting markedly more frequent use (*M* = 2.44, *SD* = 2.30) than those with ME/CFS (*M* = 1.01, *SD* = 1.86), F (1, 817) = 86.34, *p* < 0.001, partial η^2^ = 0.01. A similar pattern was found for anxiolytics (depression: *M* = 1.68, *SD* = 2.19; ME/CFS: *M* = 0.73, *SD* = 1.65), F (1, 817) = 47.26, *p* < 0.001, η^2^ = 0.06. Conversely, ME/CFS participants reported greater use of opioid antagonists (*M* = 0.72, *SD* = 1.52) than participants with depression (*M* = 0.05, *SD* = 0.32), F (1, 817) = 47.26, *p* < 0.001, η^2^ = 0.06. Further significant but smaller group differences emerged for amphetamines (depression: *M* = 0.31, ME/CFS: *M* = 0.07), cortisone (ME/CFS: *M* = 0.61 vs. depression: *M* = 0.23), painkillers (ME/CFS: *M* = 1.83 vs. depression: *M* = 1.39), psychostimulants, opioids, and virustatics (all *p* < 0.05, η^2^ = 0.006–0.022). Sleep medication was the only treatment without a statistically significant group difference, F (1, 817) = 0.51, *p* = 0.474, partial η^2^ = 0.00, suggesting comparable use across both diagnostic groups. An overview of these results is provided in Table 4.

In addition, participants with ME/CFS were particularly likely to mention low-dose naltrexone (LDN), aripiprazole, and pyridostigmine in the open-response format. Within the depression group, the open-ended responses mainly included various antidepressants, such as sertraline, duloxetine, and mirtazapine.

### 3.4. Perceived Helpfulness of Medication

**Figure 4:**
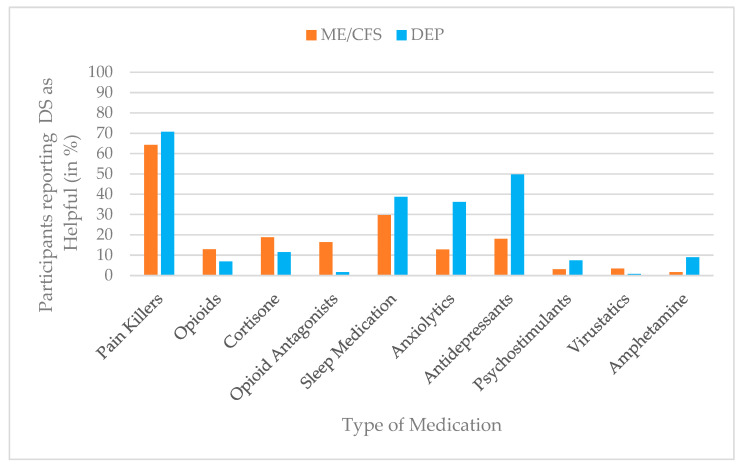
Prevalence of medication helpfulness (%) by group (ME/CFS vs. depression), based on dichotomized helpfulness ratings (helpful vs. not helpful).

For medication, painkillers were most frequently rated as helpful in both groups (ME/CFS = 64%; depression: 71%). Participants with depression more often reported helpfulness for antidepressants (depression = 50%; ME/CFS = 18%), anxiolytics (depression = 36%; ME/CFS = 13%), and sleep medication (depression = 39%; ME/CFS = 30%), whereas participants with ME/CFS more frequently reported helpfulness for opioid antagonists (ME/CFS = 16%; depression = 2%) and cortisone (ME/CFS = 19%; depression = 12%). Ratings for psychostimulants, virustatics, and amphetamines remained low in both groups (≤9%). Figure 4 summarizes the perceived helpfulness of medication across groups.

A MANOVA revealed a statistically significant multivariate group difference in the perceived helpfulness of medications between ME/CFS and depression participants, F (10, 808) = 21.85, *p* < 0.001, Pillai’s trace = 0.213, partial η^2^ = 0.21. Although Box’s M test was significant (*M* = 808.60, F (55, 761,859) = 14.46, *p* < 0.001), the robustness of Pillai’s trace supports the interpretability of the results.

Univariate follow-up ANOVAs showed the largest group differences for antidepressants, with participants in the depression group rating them as substantially more helpful (*M* = 2.93, *SD* = 3.02) than those with ME/CFS (*M* = 1.10, *SD* = 2.26), F (1, 817) = 90.34, *p* < 0.001, partial η^2^ = 0.10. A similar pattern emerged for anxiolytics (depression: *M* = 1.42, *SD* = 2.01; ME/CFS: *M* = 0.55, *SD* = 1.55), F (1, 817) = 57.39, *p* < 0.001, partial η^2^ = 0.07. Conversely, participants with ME/CFS (*M* = 0.64, *SD* = 1.40) perceived opioid antagonists as more helpful compared to participants with depression (*M* = 0.06, *SD* = 0.49), F (1, 817) = 38.89, *p* < 0.001, partial η^2^ = 0.05. Additional significant, though smaller, group differences were found for the perceived helpfulness of amphetamines (depression: *M* = 0.33, *SD* = 1.08; ME/CFS: *M* = 0.06, *SD* = 0.48), cortisone (ME/CFS: *M* = 0.74, *SD* = 1.46; depression: *M* = 0.47, *SD* = 1.28), painkillers (ME/CFS: *M* = 2.42, *SD* = 1.87; depression: *M* = 2.91, *SD* = 1.98), sleep medication (ME/CFS: *M* = 1.13, *SD* = 1.76; depression: *M* = 1.53, *SD* = 1.96), opioids (ME/CFS: *M* = 1.31, *SD* = 3.08; depression: *M* = 0.72, *SD* = 2.40), and psychostimulants (ME/CFS: *M* = 0.13, *SD* = 0.67; depression: *M* = 0.26, *SD* = 0.96; all *p* < 0.05, η^2^ = 0.007–0.029). Only virustatics showed no statistically significant group difference (ME/CFS: *M* = 0.14, *SD* = 0.69; depression: *M* = 0.05, *SD* = 0.42; *p* = 0.06), indicating comparable perceived helpfulness across both groups. For a more detailed presentation of the results, please refer to Table 5.

### 3.5. Use of Dietary Supplements Advertised for Fatigue Reduction

**Figure 5:**
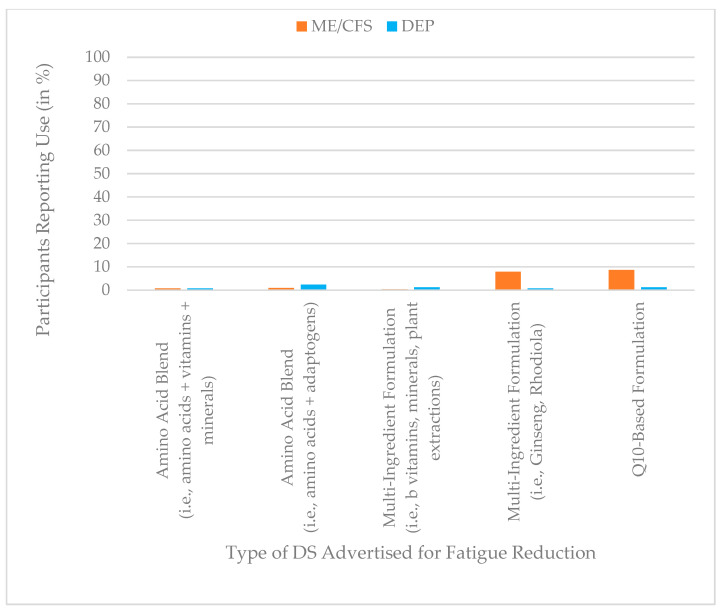
Prevalence of DS advertised for fatigue reduction use (%) by group (ME/CFS vs. depression), based on dichotomized use ratings (use vs. no use).

Use of DS advertised for fatigue reduction was generally low in both groups, with prevalence values below 10% across all categories. Among participants with ME/CFS, the highest use was reported for Q10-based formulations (9%) and multi-ingredient formulations containing ginseng or rhodiola (9%), whereas corresponding use in the depression group was markedly lower (1% for both). All other supplement categories were reported by ≤2% of participants in both groups. Reported use of DS advertised for fatigue reduction is depicted in Figure 5.

A MANOVA indicated a statistically significant multivariate group difference in use of DS advertised for fatigue reduction, F (5, 813) = 6.29, *p* < 0.001, Pillai’s trace = 0.037, partial η^2^ = 0.04, reflecting a small effect size.

Follow-up univariate analyses showed significant group differences specifically for multi-ingredient formulations combining especially Ginseng and Rhodiola, and coenzyme Q10-based products, both of which were used more frequently by ME/CFS participants (partial η^2^ = 0.015 and 0.012, respectively). The remaining categories, including both amino acid blends and other multi-ingredient formulations (especially combining B vitamins, minerals, and plant extracts), were used at comparably low levels in both groups without significant group differences (all *p* > 0.05, partial η^2^ ≤ 0.003). A summary of these results is provided in Table 6.

Additional DS advertised for fatigue reduction were not mentioned by participants at this point.

### 3.6. Perceived Helpfulness of Dietary Supplements Advertised for Fatigue Reduction

**Figure 6:**
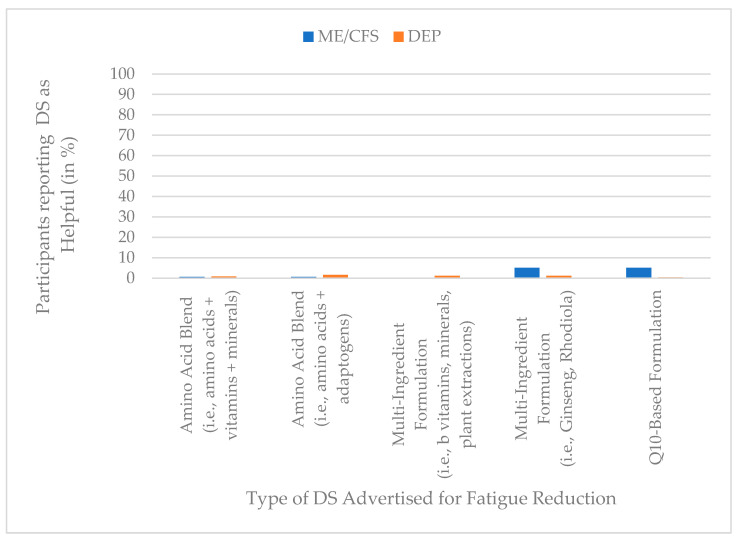
Prevalence of DS advertised for fatigue reduction helpfulness (%) by group (ME/CFS vs. depression), based on dichotomized helpfulness ratings (helpful vs. not helpful).

Perceived helpfulness of DS advertised for fatigue reduction was low overall, with all categories reported by fewer than 6% of participants in both groups. Among participants with ME/CFS, the highest helpfulness ratings were observed for Q10-based formulations (5%) and multi-ingredient formulations containing ginseng or rhodiola (5%), whereas corresponding values in the depression group were lower (0.4% and 1%, respectively). All other supplement categories were rated as helpful by ≤1.6% of participants across groups. Figure 6 presents an overview of the helpfulness of DS advertised for fatigue reduction.

A MANOVA revealed a statistically significant multivariate group difference for perceived helpfulness, F (5, 813) = 5.53, *p* < 0.001, Pillai’s trace = 0.033, partial η^2^ = 0.03, indicating a small effect. Although Box’s M test indicated a violation of covariance homogeneity (*M* = 1481.30, F (15, 916,557) = 97.94, *p* < 0.001), the robustness of Pillai’s trace supports the validity of the results.

Univariate follow-up analyses showed significant group differences for coenzyme Q10-based products, which were rated as more helpful by ME/CFS participants (partial η^2^ = 0.02), and for multi-ingredient formulations combining particularly Ginseng and Rhodiola (partial η^2^ = 0.010). The remaining DS categories, including both amino acid blends and other multi-ingredient formulations (especially combining B vitamins, minerals, and plant extracts), were rated as largely unhelpful in both groups, with no significant differences (all *p* > 0.05, partial η^2^ ≤ 0.003). A full overview of results is presented in Table 7.

### 3.7. Use of Dietary Supplements

**Figure 7:**
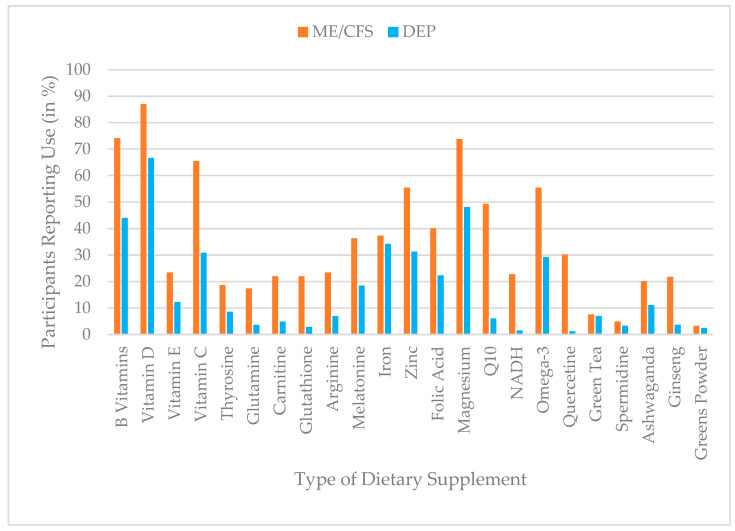
Prevalence of DS use (%) by group (ME/CFS vs. depression), based on dichotomized use ratings (use vs. no use).

Participants with ME/CFS reported higher prevalence rates of DS use across most substances compared to participants with depression. The most frequently used supplements in the ME/CFS group were vitamin D (87%), B vitamins (74%), magnesium (74%), vitamin C (66%), and omega-3 fatty acids (55%), whereas in the depression group vitamin D (67%), magnesium (48%), B vitamins (44%), iron (34%), and zinc (31%) were reported most often. Marked differences were observed for coenzyme Q10 (ME/CFS = 49%; depression = 6%), NADH (ME/CFS = 23%; depression = 2%), and quercetin (ME/CFS = 30%; depression = 1%). An overview of DS use prevalence is shown in Figure 7.

A MANCOVA was conducted to examine whether participants with ME/CFS and depression differ in their use of various DS, while statistically controlling for age, gender (male/female), and the presence of a medical recommendation for DS use. The multivariate analysis yielded a large, statistically significant effect for group, Pillai’s trace = 0.207, F (23, 551) = 6.27, *p* < 0.001, partial η^2^ = 0.21, indicating substantial overall group differences in DS use. Additionally, age (F (23, 551) = 4.12, *p* < 0.001, Pillai’s trace = 0.147, partial η^2^ = 0.15) and medical recommendation (F (23, 551) = 1.92, *p* = 0.007, Pillai’s trace = 0.074, partial η^2^ = 0.07) were significant covariates, while gender was not (F (23, 551) = 1.37, *p* = 0.116, Pillai’s trace = 0.05).

Follow-up univariate ANOVA (group effect) showed that across a wide range of DS, participants with ME/CFS reported significantly higher use than participants with depression. The strongest group differences (partial η^2^ ≥ 0.03) were found for the following substances: Q10, vitamin C, quercetin, magnesium, omega-3, melatonin, NADH, zinc, vitamin D, glutathione, and B vitamins. A detailed summary of the ANOVA results is presented in Table 8.

In addition to group differences, the covariates revealed the following effects: Medical recommendation significantly predicted higher use of magnesium (F (1, 573) = 8.92, *p* = 0.003, η^2^ = 0.02), folic acid (F (1, 573) = 7.31, *p* = 0.007, η^2^ = 0.01), arginine (F (1, 573) = 7.06, *p* = 0.008, η^2^ = 0.01), vitamin E (F (1, 573) = 6.60, *p* = 0.010, η^2^ = 0.01), vitamin C (F (1, 573) = 5.97, *p* = 0.015, η^2^ = 0.01), and B vitamins (F (1, 573) = 4.60, *p* = 0.032, η^2^ = 0.01), with greater intake reported among participants who initiated use without medical guidance. Age was a significant predictor for the use of vitamin D (F (1, 573) = 15.76, *p* < 0.001, η^2^ = 0.03), iron (F (1, 573) = 11.68, *p* < 0.001, η^2^ = 0.02), melatonin (F (1, 573) = 7.99, *p* = 0.005, η^2^ = 0.01), B vitamins (F (1, 573) = 8.04, *p* = 0.005, η^2^ = 0.01), quercetin (F (1, 573) = 5.93, *p* = 0.015, η^2^ = 0.01), spermidine (F (1, 573) = 5.60, *p* = 0.018, η^2^ = 0.01), and greens powder (F (1, 573) = 5.87, *p* = 0.016, η^2^ = 0.01), with higher use generally reported among younger participants. Table S1 presents a comprehensive summary of the detailed results.

A wide range of DS was also reported by participants in an open-response format. Amino acid complexes and turmeric for ME/CFS, and probiotics, as well as gamma-aminobutyric acid (GABA) for depression, were mentioned particularly frequently.

### 3.8. Perceived Helpfulness of Dietary Supplements

**Figure 8:**
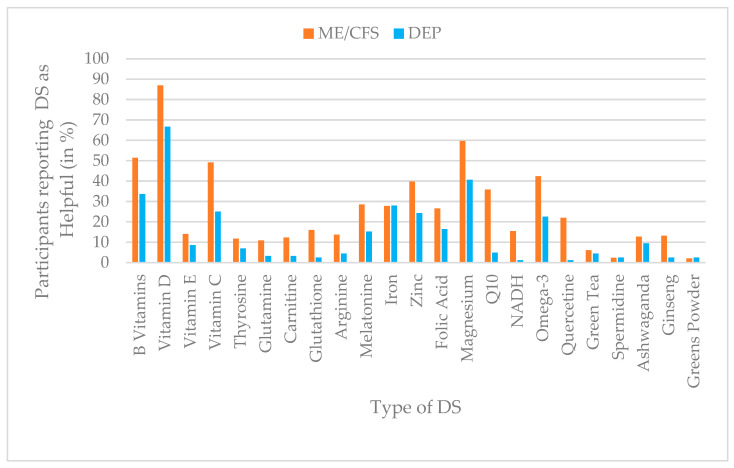
Prevalence of DS helpfulness (%) by group (ME/CFS vs. depression), based on dichotomized helpfulness ratings (helpful vs. not helpful).

Perceived helpfulness of DS was reported more frequently in the ME/CFS group across most substances, with the highest percentages for vitamin D (87%), magnesium (60%), B vitamins (51%), vitamin C (49%), and omega-3 fatty acids (42%). In the depression group, vitamin D (67%), magnesium (41%), B vitamins (34%), iron (28%), and omega-3 (23%) were most commonly rated as helpful. For several supplements, including Q10 (ME/CFS = 36%; depression = 5%), NADH (ME/CFS = 16%; depression = 1%), and quercetin (ME/CFS = 22%; depression = 1%), markedly higher helpfulness percentages were reported by participants with ME/CFS compared to those with depression. Figure 8 summarizes DS helpfulness across groups.

To examine group differences in the perceived helpfulness of the aforementioned DS while controlling for age, gender (male/female), and medical recommendation, a MANCOVA was conducted. The assumption of equality of covariance matrices was violated (Box’s M = 1470.83, F (276, 121,099) = 4.91, *p* < 0.001). However, the multivariate analysis revealed a statistically significant group effect, Pillai’s trace = 0.171, F (23, 551) = 4.95, *p* < 0.001, partial η^2^ = 0.17, indicating medium-sized overall differences in perceived helpfulness across the two diagnostic groups. In addition, significant multivariate effects were found for age (Pillai’s trace = 0.126, F (23, 551) = 3.45, *p* < 0.001, partial η^2^ = 0.13), medical recommendation (Pillai’s trace = 0.073, F (23, 551) = 1.90, *p* = 0.007, partial η^2^ = 0.07), and gender (Pillai’s trace = 0.070, F (23, 551) = 1.80, *p* = 0.013, partial η^2^ = 0.07).

Follow-up univariate ANOVAs for group differences showed that participants with ME/CFS rated the following DS as significantly more helpful than those with depression: Q10, quercetin, omega-3, vitamin C, NADH, magnesium, glutathione, melatonin, carnitine, arginine, ginseng, zinc, glutamine, and folic acid. The largest group effects were observed for Q10 (partial η^2^ = 0.081), quercetin (partial η^2^ = 0.064), and omega-3 (partial η^2^ = 0.037). No significant group differences emerged for vitamin D, vitamin E, tyrosine, spermidine, ashwagandha, iron, greens powder, or green tea (*p* > 0.05). The results are presented in detail in Table 9.

In addition, perceived helpfulness ratings were significantly predicted by other covariates: Medical recommendation significantly predicted ratings for magnesium (F (1, 573) = 12.81, *p* < 0.001, η^2^ = 0.02), arginine (F (1, 573) = 10.43, *p* = 0.001, η^2^ = 0.02), omega-3 (F (1, 573) = 6.11, *p* = 0.014, η^2^ = 0.01), ashwagandha (F (1, 573) = 6.10, *p* = 0.014, η^2^ = 0.01), and vitamin E (F (1, 573) = 4.42, *p* = 0.036, η^2^ = 0.01), with higher helpfulness reported when DS were taken without medical recommendation. Age significantly predicted ratings for melatonin (F (1, 573) = 17.36, *p* < 0.001, η^2^ = 0.03), iron (F (1, 573) = 21.14, *p* < 0.001, η^2^ = 0.04), glutathione (F (1, 573) = 10.11, *p* = 0.002, η^2^ = 0.02), quercetin (F (1, 573) = 6.15, *p* = 0.013, η^2^ = 0.01), and greens powder (F (1, 573) = 7.36, *p* = 0.007, η^2^ = 0.01), with younger participants giving higher helpfulness ratings. Gender yielded small but significant effects (η^2^ = 0.007–0.011) for vitamin D (F (1, 573) = 4.21, *p* = 0.041, η^2^ = 0.007), carnitine (F (1, 573) = 4.57, *p* = 0.033, η^2^ = 0.008), arginine (F (1, 573) = 6.58, *p* = 0.011, η^2^ = 0.011), and zinc (F (1, 573) = 5.82, *p* = 0.016, η^2^ = 0.010), with female participants consistently reporting higher perceived helpfulness. Further details of all results are available in Table S1.

## 4. Discussion

The present study examined group differences between participants self-identifying with ME/CFS or depression in terms of their use and perceived helpfulness of a broad range of behavioral, psychological, pharmacological, and DS interventions. By combining prevalence estimates with multivariate analyses based on continuous ratings, the study provides a detailed picture of real-world treatment engagement across two conditions that share substantial symptom overlap but differ markedly in diagnostic recognition, treatment availability, and clinical guidance.

### 4.1. Group Differences in Intervention Use and Perceived Helpfulness

Robust group differences emerged between ME/CFS and depression across nearly all examined domains. Participants with depression showed a comparatively structured and guideline-concordant pattern of intervention use [34,35], with high prevalence rates for psychotherapy (78%) and antidepressant medication (50%). These findings are consistent with epidemiological and clinical data indicating that psychotherapy and antidepressants represent first-line treatments for depression and are widely implemented within routine care pathways [34,35,69]. Meta-analyses of randomized controlled trials report small-to-moderate effect sizes for antidepressant medication (g = 0.24–0.30) [36] and moderate effects for psychotherapy, particularly CBT (g = 0.56–0.63) [37], supporting their central role in clinical practice.

In contrast, participants with ME/CFS reported substantially more heterogeneous intervention patterns. Prevalence estimates indicated particularly high engagement in pacing (over 90%), dietary supplements (approximately 70%), relaxation or meditation techniques (approximately 80%), and dietary changes (approximately 50%). This distribution closely aligns with current clinical recommendations that emphasize pacing as a core management strategy for ME/CFS in the absence of curative or disease-modifying treatments [57]. At the same time, the high prevalence of complementary and self-directed strategies might reflect the limited availability of standardized, evidence-based treatment protocols and restricted access to specialized care for ME/CFS [24].

Importantly, although many interventions were frequently used, perceived helpfulness ratings were generally modest in both groups. Even for commonly applied strategies, only a minority of interventions reached higher helpfulness prevalence thresholds, underscoring the ongoing therapeutic uncertainty experienced by patients with both conditions [21,22]. These findings mirror prior research in ME/CFS populations, which consistently reports extensive trial-and-error treatment behavior combined with limited perceived benefit [24,70,71].

### 4.2. Medication Use: Alignment with Clinical Pathways vs. Experimental Approaches

Medication further highlighted structural differences between the two diagnostic groups. Participants with depression reported higher prevalence of use and perceived helpfulness of conventional psychopharmacotherapeutics, including antidepressants, anxiolytics, and mood stabilizers, consistent with established psychopharmacological treatment algorithms and reimbursement structures [34,35].

In contrast, participants with ME/CFS more frequently reported the use of medications outside standard psychiatric pathways, including cortisone and opioid antagonists. In particular, low-dose naltrexone (LDN) was frequently mentioned in open-text responses, reflecting growing patient interest despite limited and inconclusive clinical trial evidence [15,16,72]. These findings align with emerging literature describing off-label medication use in ME/CFS as a consequence of limited therapeutic options and insufficient evidence-based guidance. Retrospective observational studies have reported symptomatic improvements with agents such as low-dose aripiprazole, yet these results arise from non-randomized designs and remain exploratory rather than confirmatory [17]. Systematic reviews of randomized controlled trials in ME/CFS have not identified clearly effective pharmacological treatments, highlighting the sparse and heterogeneous nature of the evidence base and reinforcing the exploratory nature of pharmacological treatment attempts in this population [52].

### 4.3. Dietary Supplements and Products Advertised for Fatigue Symptoms: Prevalence vs. Practical Relevance

Substantial group differences were also observed in the use and perceived helpfulness of DS. Participants with ME/CFS reported markedly higher prevalence rates for supplements targeting energy metabolism and oxidative stress, including vitamin D (87%), magnesium (74%), B vitamins (74%), coenzyme Q10 (49%), and omega-3 fatty acids (55%). These patterns correspond with current hypotheses implicating mitochondrial dysfunction, immune dysregulation, and oxidative stress in ME/CFS pathophysiology [73,74,75].

However, despite statistically significant group differences, absolute mean levels and prevalence of perceived helpfulness remained moderate to low for most supplements. This observation is consistent with systematic reviews and meta-analyses indicating limited and inconsistent evidence for the efficacy of most supplements in ME/CFS, often based on small samples and heterogeneous outcome measures [49,51]. Similarly, supplements specifically marketed for fatigue reduction showed very low prevalence of use and minimal perceived helpfulness in both groups, suggesting limited clinical relevance despite commercial availability.

Beyond questions of efficacy, the observed breadth of intervention use raises important clinical safety considerations. Potential polypharmacological effects present considerable clinical concern in the treatment landscape of both ME/CFS and depression [76,77]. Given the frequent co-administration of multiple DS and medications, often without consistent medical supervision, there is a heightened risk for pharmacodynamic and pharmacokinetic interactions [78,79]. These may not only attenuate the intended therapeutic effects but could also contribute to symptom exacerbation or new side effects, particularly in a patient population already characterized by physiological vulnerability [79].

These findings emphasize the importance of interpreting statistical significance alongside absolute prevalence and effect magnitude. While supplement use is widespread, particularly among individuals with ME/CFS, the practical benefits appear limited, highlighting a gap between patient behavior, marketing claims, and empirical evidence.

### 4.4. Contextual and Psychosocial Factors Shaping Treatment Engagement

Beyond diagnostic group differences, treatment engagement was shaped by contextual and personal factors. Younger age and self-initiated use without medical recommendation were associated with higher supplement intake and perceived helpfulness, echoing previous findings that autonomy, expectancy effects, and health-related beliefs influence treatment perceptions [80,81,82]. Similar subgroup patterns, like stronger responses among patients with fatigue or orthostatic symptoms, have been reported by Eckey et al. [83], underscoring heterogeneity in DS responses and the need for subgroup-based analyses. Illness representations and diagnostic labeling processes may further contribute to these patterns [84,85,86]. Individuals identifying with ME/CFS often conceptualize their condition as a systemic, somatic, and energy-related disorder, increasing the perceived plausibility of biologically oriented and metabolism-targeted interventions [28,87]. In contrast, individuals with depression may adopt a biopsychosocial illness model that aligns more closely with psychotherapy and pharmacotherapy, reinforcing adherence to guideline-based care [88,89]. Prior research on labeling effects and illness identity suggests that such frameworks strongly influence treatment expectations, engagement, and perceived helpfulness across chronic and stigmatized conditions [86,90,91,92,93].

At the same time, partial overlap in treatment use and perceived helpfulness across groups warrants careful interpretation. The observed similarities in treatment responses might reflect not only possibly shared pathophysiological mechanisms (e.g., mitochondrial dysfunction, oxidative stress) but also unrecognized diagnostic comorbidity [29]. Given the self-reported nature of group assignment, undetected depressive symptoms in the ME/CFS group may partly account for the perceived efficacy of depression treatments [18,94,95,96,97]. While this overlap highlights the relevance of integrative treatment frameworks addressing converging biological and psychosocial pathways, it also calls for caution, as part of the apparent similarity in treatment responses may reflect diagnostic overlap rather than true pathophysiological convergence.

### 4.5. Limitations

The present findings should be interpreted descriptively rather than causally. Reported use and perceived helpfulness do not constitute evidence of treatment efficacy. In particular, placebo and expectancy effects may contribute substantially to perceived benefit, especially in conditions characterized by diagnostic uncertainty and limited treatment options [77,78,79].

Several limitations warrant consideration. First, group assignment for both ME/CFS and depression was based on self-reported diagnoses and was not clinically verified. While this approach allowed for broad recruitment, it may have introduced diagnostic heterogeneity. To partially address this, the ME/CFS cardinal criterion PEM was systematically assessed using the DSQ-PEM, providing additional symptom-based characterization of the ME/CFS group. Similarly, depressive symptom severity was assessed using DASS-21. Nevertheless, misclassification cannot be fully excluded, particularly in light of symptom overlap and potential subclinical presentations. Second, the self-reported nature of treatment and intervention use, as well as perceived helpfulness, is subject to recall and response biases. In addition, it was not specified whether the reported medications or DS were taken for the respective condition or for unrelated health issues. No data was collected on dosage, duration, or formulation of the reported interventions, limiting the interpretability of the findings. Additionally, the present study did not assess concurrent intake patterns or combinations of substances, representing a significant limitation in the interpretation of potential compound-related effects. Treatment and intervention effects may vary significantly depending on these parameters. Future studies should therefore record indication context and treatment parameters and compare diagnostically matched groups receiving comparable interventions under standardized conditions. Despite these limitations, the large sample size and systematic assessment of both prevalence and perceived helpfulness provide valuable insight into real-world treatment behavior across ME/CFS and depression.

## 5. Conclusions

In summary, this study demonstrated pronounced differences in intervention strategies between individuals self-identifying with ME/CFS or depression. Depression was characterized by a high prevalence of structured, guideline-based interventions, whereas ME/CFS was associated with diverse, often self-directed treatment portfolios extending beyond conventional care. Despite widespread use, most interventions were perceived as only modestly helpful, underscoring substantial unmet therapeutic needs in both conditions, particularly in ME/CFS. Future research should integrate longitudinal designs, objective treatment data, and controlled trials to better delineate effective interventions. Taken together, the findings point to a need for further development and evaluation of empirically supported, patient-centered treatment and intervention strategies for ME/CFS and suggest differences in clinical care structures between ME/CFS and depression.

## Figures and Tables

**Table 1:** Sociodemographic data for the ME/CFS and depression groups.

Variables	ME/CFS *n* = 576	Depression *n* = 243
Gender *		
Female	514 (89.2%)	189 (77.7%)
Male	58 (10.1%)	45 (18.6%)
Non-Binary	4 (0.7%)	9 (3.7%)
Age in years *		
*M* and *SD*	*M* = 46.37 (*SD* = 11.68)	*M* = 38.37 (*SD* = 13.54)
Range	18–72	18–75
Education *		
No school diploma	3 (0.5%)	4 (1.6%)
Compulsory education	19 (3.3%)	19 (7.8%)
Apprenticeship	139 (24.3%)	45 (18.5%)
High school diploma	125 (21.9%)	74 (30.5%)
University degree	220 (38.2%)	83 (34.2%)
Other	65 (11.4%)	18 (7.4%)
BMI		
*M* and *SD*	*M* = 25.84 (*SD* = 5.81)	*M* = 25.78 (*SD* = 6.86)
Range	14.4–53.28	14.36–57.37
Fatigue symptom duration (in months)		
*M*, Mdn and *SD*	*M* = 59.38; Mdn = 31.00; *SD* = 67.95	*M* = 49.96; Mdn = 26.99; *SD* = 59.35
Range	1–350	1–312

* indicates a statistically significant difference between the groups (*p* < 0.001).

**Table 2:** Univariate ANOVA results for group differences in intervention use based on mean Likert-scale ratings (0–5).

Measure	ME/CFS		DEP		F (1, 817)	*p*	η^2^
	*M*	*SD*	*M*	*SD*			
Pacing	2.73	0.8	0.16	0.62	2022.89	<0.001	0.71
Dietary Supplementation	2.43	1.09	1.22	1.34	184.14	<0.001	0.18
Breathing therapy	1.66	1.36	0.74	1.08	87.66	<0.001	0.09
Dietary Changes	1.62	1.39	0.79	1.21	64.67	<0.001	0.07
Physiotherapy	2.02	1.24	1.28	1.31	57.31	<0.001	0.07
Relaxation/Meditation	2.4	1.04	1.81	1.15	51.85	<0.001	0.06
Psychotherapy	1.81	1.34	2.49	0.99	51.17	<0.001	0.06
Homeopathy	1.04	1.28	0.57	1.02	26.45	<0.001	0.03
Acupuncture	0.87	1.19	0.54	0.95	14.80	<0.001	0.02
Mindfulness therapy	1.88	1.31	1.54	1.2	12.04	<0.001	0.02
Art therapy	0.44	0.93	0.69	1.05	11.43	<0.001	0.01
Magnet therapy	0.25	0.72	0.09	0.41	10.95	<0.001	0.01
Rehab	1.15	1.21	0.85	1.19	10.49	0.001	0.01
Medication	1.88	1.35	2.2	1.26	10.06	0.002	0.01
Fasting	0.31	0.79	0.16	0.58	6.72	0.010	0.01
Spiritual practices	0.44	0.95	0.31	0.82	3.12	0.078	0.00
Exercise therapy	1.22	1.29	1.42	1.34	4.20	0.041	0.01
Ergotherapy	1.16	1.34	0.94	1.22	4.91	0.27	0.01
Aromatherapy	0.47	0.99	0.49	0.96	0.05	0.817	0.00

**Table 3:** Univariate ANOVA Results for group differences in intervention helpfulness based on mean Likert-scale ratings (0–5).

Measure	ME/CFS		DEP		F (1, 817)	*p*	η^2^
	*M*	*SD*	*M*	*SD*			
Pacing	3.50	1.22	0.22	0.90	1423.71	<0.001	0.64
Dietary Supplementation	1.92	1.93	0.99	1.66	184.14	<0.001	0.18
Medication	1.47	1.90	2.59	1.76	66.19	<0.001	0.08
Psychotherapy	2.08	1.90	3.14	1.63	57.15	<0.001	0.07
Exercise therapy	1.02	1.46	1.92	1.95	52.77	<0.001	0.06
Breathing therapy	2.01	1.89	1.09	1.71	43.04	<0.001	0.05
Dietary Changes	1.82	1.93	0.88	1.66	42.03	<0.001	0.05
Art therapy	0.53	1.26	1.12	1.78	28.90	<0.001	0.03
Physiotherapy	6.09	3.76	4.28	4.21	21.77	<0.001	0.03
Relaxation/Meditation	3.03	1.62	2.50	1.87	16.71	<0.001	0.02
Acupuncture	1.01	1.59	0.60	1.26	12.48	<0.001	0.02
Homeopathy	2.09	1.87	2.56	1.80	11.25	<0.001	0.01
Magnetic field therapy	0.27	0.96	0.08	0.51	8.53	0.004	0.01
Fasting	0.38	1.12	0.23	0.87	3.65	0.057	0.00
Aromatherapy	0.54	1.311	0.73	1.52	3.14	0.077	0.00
Mindfulness therapy	2.40	1.91	2.21	1.96	1.68	0.195	0.00
Spiritual practices	0.48	1.21	0.39	1.11	1.07	0.302	0.00
Rehab	1.19	1.53	1.33	1.87	1.04	0.309	0.00
Ergotherapy	1.32	1.80	1.27	1.78	0.13	0.714	0.00

**Table 4:** Univariate ANOVA results for group differences in medication use based on mean Likert-scale ratings (0–5).

Measure	ME/CFS		DEP		F (1, 817)	*p*	η^2^
	*M*	*SD*	*M*	*SD*			
Antidepressants	1.01	1.86	2.44	2.30	86.34	<0.001	0.10
Anxiolytics	0.73	1.62	1.68	2.19	47.26	<0.001	0.06
Opioid antagonists	0.72	1.52	0.05	0.32	47.25	<0.001	0.05
Amphetamine	0.07	0.54	0.07	0.54	18.28	<0.001	0.02
Cortisone	0.61	1.32	0.23	0.72	17.80	<0.001	0.02
Painkillers	1.83	1.96	1.39	1.54	9.86	0.002	0.01
Psychostimulants	0.15	0.78	0.32	1.13	5.83	0.016	0.01
Opioids	0.45	1.27	0.24	0.91	5.41	0.020	0.01
Virustatics	0.09	0.52	0.02	0.17	5.08	0.024	0.01
Sleep medication	1.05	1.79	1.14	1.73	0.51	0.474	0.00

**Table 5:** Univariate ANOVA Results for group differences in medication helpfulness based on mean Likert-scale ratings (0–5).

Measure	ME/CFS		DEP		F (1, 817)	*p*	η^2^
	*M*	*SD*	*M*	*SD*			
Antidepressants	1.10	2.26	2.93	3.02	90.34	<0.001	0.10
Anxiolytics	0.55	1.32	1.42	1.88	57.39	<0.001	0.07
Opioid antagonists	0.64	140	0.06	0.49	38.89	<0.001	0.05
Amphetamine	0.06	0.48	0.33	1.08	24.39	<0.001	0.03
Painkillers	2.42	1.87	2.91	1.98	11.49	<0.001	0.01
Sleep medication	1.13	1.76	1.53	1.96	8.24	0.004	0.01
Opioids	1.31	3.08	0.72	2.40	6.94	0.009	0.01
Cortisone	0.74	1.46	0.47	1.28	6.09	0.014	0.01
Psychostimulants	0.13	0.67	0.26	0.96	5.57	0.019	0.01
Virustatics	0.14	0.69	0.05	0.42	3.61	0.058	0.00

**Table 6:** Univariate ANOVA results for group differences in DS advertised for fatigue symptoms use based on mean Likert-scale ratings (0–5).

Measure	ME/CFS		DEP		F (1, 817)	*p*	η^2^
	*M*	*SD*	*M*	*SD*			
Multi-Ingredient (i.e., Ginseng, Rhodiola)	0.26	0.92	0.05	0.42	12.02	<0.001	0.02
Q10-based	0.17	0.72	0.02	0.29	9.58	0.002	0.01
Amino acid blend (i.e., amino acids + adaptogens)	0.01	0.15	0.03	0.31	2.24	0.135	0.00
Multi-ingredient formulation (i.e., B vitamins, minerals, plant extracts)	0.03	0.34	0.07	0.52	1.91	0.167	0.00
Amino acid blend (i.e., amino acids + vitamins & minerals)	0.03	0.32	0.03	0.32	0.01	0.977	0.00

**Table 7:** Univariate ANOVA results for group differences in ds advertised for fatigue symptoms helpfulness based on mean Likert-scale ratings (0–5).

Measure	ME/CFS		DEP		F (1, 817)	*p*	η^2^
	*M*	*SD*	*M*	*SD*			
Q10-based	0.19	0.75	0.02	0.21	13.25	<0.001	0.02
Multi-Ingredient (i.e., Ginseng, Rhodiola)	0.26	0.92	0.05	0.42	8.57	0.004	0.01
Multi-ingredient formulation (i.e., B vitamins, minerals, plant extracts)	0.03	0.32	0.08	0.51	2.88	0.090	0.00
Amino acid blend (i.e., amino acids + adaptogens)	0.02	0.42	0.10	0.93	2.79	0.095	0.00
Amino acid blend (i.e., amino acids + vitamins & minerals)	0.03	0.32	0.04	0.45	0.29	0.587	0.00

**Table 8:** Univariate ANOVA results for group differences in DS use based on mean Likert-scale ratings (0–5).

Measure	ME/CFS		DEP		F (1, 573)	*p*	η^2^
	*M*	*SD*	*M*	*SD*			
Q10	2.13	2.07	0.28	0.91	82.02	<0.001	0.13
Vitamin C	2.88	2.07	1.28	1.79	52.61	<0.001	0.08
Quercetin	1.17	1.80	0.07	0.45	44.79	<0.001	0.07
Magnesium	3.30	1.98	1.93	1.95	40.08	<0.001	0.07
Omega-3	2.41	2.14	1.05	1.65	39.94	<0.001	0.07
Melatonin	1.38	1.97	0.52	1.24	23.89	<0.001	0.04
NADH	0.81	1.53	0.07	0.45	22.99	<0.001	0.04
Zinc	2.38	2.11	1.28	1.69	21.39	<0.001	0.04
Vitamin D	4.12	1.46	3.22	1.77	20.29	<0.001	0.03
Glutathione	0.72	1.42	0.18	0.84	17.65	<0.001	0.03
B Vitamins	3.14	1.92	2.18	1.91	17.03	<0.001	0.03
Ginseng	0.72	1.43	0.16	0.66	12.75	<0.001	0.02
Carnitine	0.77	1.49	0.27	1.06	11.92	<0.001	0.02
Arginine	0.78	1.47	0.29	1.06	11.28	<0.001	0.02
Glutamine	0.62	1.37	0.22	0.93	8.76	0.003	0.02
Folic acid	1.65	2.02	1.03	1.70	7.52	0.006	0.01
Vitamin E	1.07	1.82	0.63	1.37	5.01	0.026	0.01
Iron	1.47	1.92	1.28	1.69	2.34	0.127	0.00
Ashwagandha	0.62	1.33	0.42	1.09	1.94	0.164	0.00
Spermidine	0.12	0.57	0.17	0.70	1.83	0.177	0.00
Tyrosine	0.74	1.58	0.54	1.32	1.37	0.242	0.00
Greens powder	0.12	0.65	0.11	0.61	0.35	0.557	0.00
Green tea	0.29	1.03	0.27	0.92	0.06	0.8	0.00

**Table 9:** Univariate ANOVA results for group differences in ds helpfulness based on mean Likert-scale ratings (0–5).

Measure	ME/CFS		DEP		F (1, 573)	*p*	η^2^
	*M*	*SD*	*M*	*SD*			
Q10	1.67	1.88	0.39	1.22	50.18	<0.001	0.08
Quercetin	1.03	1.70	0.08	0.511	38.87	<0.001	0.06
Omega-3	1.91	1.90	1.04	1.73	22.21	<0.001	0.04
NADH	0.71	1.40	0.09	0.62	21.66	<0.001	0.04
Magnesium	2.61	1.79	1.84	1.89	18.27	<0.001	0.03
Glutathione	0.74	1.52	0.19	0.91	18.20	<0.001	0.03
Melatonin	1.22	1.76	0.61	1.32	18.16	<0.001	0.03
Vitamin C	2.26	1.92	1.42	2.03	16.82	<0.001	0.03
Carnitine	0.90	1.97	0.28	1.25	13.15	<0.001	0.02
Arginine	0.68	1.40	0.24	0.88	12.06	<0.001	0.02
Ginseng	0.64	1.39	0.16	0.70	10.95	<0.001	0.02
Zinc	1.85	1.90	1.23	1.76	8.32	0.004	0.01
Glutamine	0.58	1.37	0.23	0.91	7.60	0.006	0.01
Folic acid	1.30	1.82	0.88	1.60	6.14	0.013	0.01
B Vitamins	2.18	1.67	1.83	1.74	2.99	0.084	0.01
Vitamin D	7.42	2.50	7.06	3.04	0.65	0.421	0.00
Greens Powder	0.11	0.67	0.11	0.61	0.46	0.497	0.00
Vitamin E	0.69	1.40	0.67	1.55	0.28	0.598	0.00
Tyrosine	0.58	1.36	0.55	1.42	0.18	0.672	0.00
Spermidine	0.13	0.64	0.15	0.65	0.15	0.701	0.00
Ashwagandha	0.61	1.38	0.56	1.43	0.12	0.732	0.00
Iron	1.26	1.77	1.41	1.89	0.01	0.915	0.00
Green tea	0.27	1.01	0.28	1.01	0.02	0.889	0.00

## Data Availability

The original data presented in the study are openly available in OSF at https://osf.io/8xv4d/, accessed on 13 January 2026.

## Supplementary Materials

The following supporting information can be downloaded at: https://www.mdpi.com/article/10.3390/jcm15020849/s1, Table S1: (M)ANOVA tables; File S1: STROBE Statement Checklist.

## Abbreviations

The following abbreviations are used in this manuscript:

ANOVA	Analysis of Variance
CAM	Complementary and Alternative Medicine
CBT	Cognitive Behavioral Therapy
CoQ10	Coenzyme Q10
DASS-21	Depression, Anxiety, and Stress Scale
DEP	Depression
DS	Dietary Supplement
DSQ-PEM	DePaul Symptom Questionnaire—Post Exertional Malaise
GABA	Gamma-Aminobutyric Acid
GET	Graded Exercise Therapy
LDN	Low-Dose Naltrexone
M	Mean
MANCOVA	Multivariate Analyses of Covariance
MANOVA	Multivariate Analyses of Variance
Mdn	Median
ME/CFS	Myalgic Encephalomyelitis/Chronic Fatigue Syndrome
NADH	Nicotinamide Adenine Dinucleotide
NICE	National Institute for Health and Care Excellence
PEM	Post-Exertional Malaise
RCT	Randomized Controlled Trial
SD	Standard Deviation
SNRI	Serotonin–Norepinephrine Reuptake Inhibitors
SSRI	Selective Serotonin Reuptake Inhibitors
