# Supplementary material for: Use and Perceived Helpfulness of Different Intervention Strategies in Myalgic Encephalomyelitis/Chronic Fatigue Syndrome and Depression
Source: J Clin Med. 2026 Jan 20;15(2):849. doi: 10.3390/jcm15020849 (PMC12842491; doi:10.3390/jcm15020849)
Supplement: Supplementary file 1 [file jcm-15-00849-s001.zip › File S1_STROBE-cross-sectional.docx]

STROBE Statement—Checklist of items that should be included in reports of ***cross-sectional studies***

|  | Item No | Recommendation | Page No. | Explanation |
| --- | --- | --- | --- | --- |
| **Title and abstract** | 1 | (*a*) Indicate the study’s design with a commonly used term in the title or the abstract | 1 | The study design (cross-sectional online survey) is indicated in the abstract. |
|  |  | (*b*) Provide in the abstract an informative and balanced summary of what was done and what was found | 1 | The abstract provides a balanced summary of the study background, methods, sample characteristics, statistical analyses, and key findings. |
| Introduction | | |  |  |
| Background/rationale | 2 | Explain the scientific background and rationale for the investigation being reported | 2-3 | The introduction outlines the clinical and scientific background of ME/CFS and depression, highlights gaps in evidence regarding treatment use and perceived helpfulness, and explains the rationale for a comparative investigation. |
| Objectives | 3 | State specific objectives, including any prespecified hypotheses | 3 | The study aimed to compare the use and perceived helpfulness of conventional, complementary, and self-directed treatments between individuals with self-reported ME/CFS and depression. Due to the exploratory nature of the study, no formal a priori hypotheses were specified. |
| Methods | | |  |  |
| Study design | 4 | Present key elements of study design early in the paper | 1;  4-5 | This study employed a cross-sectional online survey design. |
| Setting | 5 | Describe the setting, locations, and relevant dates, including periods of recruitment, exposure, follow-up, and data collection | 4 | Data were collected between May and December 2024 via an online survey administered using the SoSciSurvey platform. Recruitment took place through psychiatric and psychotherapeutic services in Lower Austria and Vienna and through online patient communities. |
| Participants | 6 | (*a*) Give the eligibility criteria, and the sources and methods of selection of participants | 4 | Eligible participants were adults aged 18–70 years with a self-reported diagnosis of either ME/CFS or depression. Participants were recruited via clinical settings and online platforms using convenience sampling. |
| Variables | 7 | Clearly define all outcomes, exposures, predictors, potential confounders, and effect modifiers. Give diagnostic criteria, if applicable | 4-5 | Primary variables included self-reported use and perceived helpfulness of interventions, medications, and dietary supplements. Diagnostic group (ME/CFS vs. depression) served as the main exposure variable. Age, gender, and medical recommendation were considered potential confounders. |
| Data sources/ measurement | 8* | For each variable of interest, give sources of data and details of methods of assessment (measurement). Describe comparability of assessment methods if there is more than one group | 4-5 | Data were collected via self-report questionnaires. Post-exertional malaise was assessed using the DSQ-PEM, and depressive symptom severity was assessed using the DASS-21. Intervention use was asked on a 5-skale likert base, and additional open-ended questions were used to gain additional information on intervention use not provided in the questions before. aAssessment methods were identical across groups. |
| Bias | 9 | Describe any efforts to address potential sources of bias | 4-5; 17-20 | Potential sources of bias included self-reported diagnoses, recall bias, and self-selection into an online survey. These were addressed through the use of validated instruments, independent recruitment for diagnostic groups, and covariate-adjusted multivariate analyses. |
| Study size | 10 | Explain how the study size was arrived at | 4 | Study size was determined by the number of participants who accessed and completed the online survey during the recruitment period. |
| Quantitative variables | 11 | Explain how quantitative variables were handled in the analyses. If applicable, describe which groupings were chosen and why | 4-5 | Likert-scale variables were analyzed as continuous mean scores in multivariate analyses. For descriptive prevalence estimates, variables were dichotomized using predefined cut-off scores. |
| Statistical methods | 12 | (*a*) Describe all statistical methods, including those used to control for confounding | 5 | Statistical analyses included descriptive statistics, MANOVA and MANCOVA to examine group differences, and adjustment for age, gender, and medical recommendation. |
|  |  | (*b*) Describe any methods used to examine subgroups and interactions | 5 | Subgroup analyses were conducted by diagnostic group. No interaction analyses were performed. |
|  |  | (*c*) Explain how missing data were addressed | 5 | Only fully completed questionnaires were included; therefore, no missing data were present in the final analytical dataset. |
|  |  | (*d*) If applicable, describe analytical methods taking account of sampling strategy | n.a. | Not applicable. The study used a convenience-based online sample without weighting or sampling-adjusted analyses. |
|  |  | (*e*) Describe any sensitivity analyses | 5;  7-20 | Sensitivity analyses were conducted by comparing unadjusted MANOVA models with covariate-adjusted MANCOVA models. |
| Results | | |  |  |
| Participants | 13* | (a) Report numbers of individuals at each stage of study—eg numbers potentially eligible, examined for eligibility, confirmed eligible, included in the study, completing follow-up, and analysed | 6 | A total of 3,637 individuals accessed the survey; 819 fully completed questionnaires were included in the final analyses. |
|  |  | (b) Give reasons for non-participation at each stage | 4 | Non-participation was primarily due to incomplete questionnaires. |
|  |  | (c) Consider use of a flow diagram | n.a. | A participant flow diagram is not provided. |
| Descriptive data | 14* | (a) Give characteristics of study participants (eg demographic, clinical, social) and information on exposures and potential confounders | 6-7 | Participant characteristics, including demographic and clinical variables, are presented in Table 1. |
|  |  | (b) Indicate number of participants with missing data for each variable of interest | 4 | No missing data were present for variables of interest in the analyzed dataset. |
| Outcome data | 15* | Report numbers of outcome events or summary measures | 4-5;  6-20 | Summary measures of intervention use and perceived helpfulness are reported as means, prevalence rates, and effect sizes. |
| Main results | 16 | (*a*) Give unadjusted estimates and, if applicable, confounder-adjusted estimates and their precision (eg, 95% confidence interval). Make clear which confounders were adjusted for and why they were included |  | Unadjusted and covariate-adjusted estimates are reported, including effect sizes and statistical significance. |
|  |  | (*b*) Report category boundaries when continuous variables were categorized | 5 | Cut-off scores used for dichotomization are reported in the Methods section. |
|  |  | (*c*) If relevant, consider translating estimates of relative risk into absolute risk for a meaningful time period | n.a. | Not applicable |
| Other analyses | 17 | Report other analyses done—eg analyses of subgroups and interactions, and sensitivity analyses | 5;  6-20 | Additional analyses included descriptive prevalence estimates. |
| Discussion | | |  |  |
| Key results | 18 | Summarise key results with reference to study objectives | 20-23 | The discussion summarizes key findings in relation to the study objectives. |
| Limitations | 19 | Discuss limitations of the study, taking into account sources of potential bias or imprecision. Discuss both direction and magnitude of any potential bias | 23 | Limitations include self-reported diagnoses, potential selection bias, and the cross-sectional design, which precludes causal inference. |
| Interpretation | 20 | Give a cautious overall interpretation of results considering objectives, limitations, multiplicity of analyses, results from similar studies, and other relevant evidence | 20-23 | Results are interpreted cautiously in light of existing literature, methodological limitations, and the exploratory nature of the analyses. |
| Generalisability | 21 | Discuss the generalisability (external validity) of the study results | 20-23 | Findings may not be generalizable beyond individuals with ME/CFS or depression who engage in online surveys and self-directed treatment strategies. |
| Other information | | |  |  |
| Funding | 22 | Give the source of funding and the role of the funders for the present study and, if applicable, for the original study on which the present article is based | 4; 24 | The sources of funding and the role of the funders are reported in the Funding section. Anyway, there was no external funding. |

*Give information separately for exposed and unexposed groups.

**Note:** An Explanation and Elaboration article discusses each checklist item and gives methodological background and published examples of transparent reporting. The STROBE checklist is best used in conjunction with this article (freely available on the Web sites of PLoS Medicine at http://www.plosmedicine.org/, Annals of Internal Medicine at http://www.annals.org/, and Epidemiology at http://www.epidem.com/). Information on the STROBE Initiative is available at www.strobe-statement.org.
